# Supplementary material for: Mechanically activated mesenchymal-derived bone cells drive vessel formation via an extracellular vesicle mediated mechanism
Source: J Tissue Eng. 2023 Aug 29;14:20417314231186918. doi: 10.1177/20417314231186918 (PMC10467237; doi:10.1177/20417314231186918)
Supplement: sj-docx-1-tej-10.1177_20417314231186918 – Supplemental material for Mechanically activated mesenchymal-derived bone cells drive vessel formation via an extracellular vesicle mediated mechanism [file sj-docx-1-tej-10.1177_20417314231186918.docx]

**Mechanically activated mesenchymal-derived bone cells drive vessel formation via an extracellular vesicle mediated mechanism**

**Key words:** Osteoblast; Osteocyte; Angiogenesis; VEGF; miRNA; miRNA-150-5p; Mechanobiology;

Shen, N.^1,2^ *, Maggio, M.^1,2^ *, Woods, I.^1,2^, Lowry, M.^3^, Almasri, R.^3^, Gorgun, C.^1,2,5^, Eichholz, K.F ^1,2^, Stavenschi E.^1,2^, Hokamp, K.^4^, Roche, F.M.^4^, O’Driscoll, L.^3^, Hoey, D.A.^1,2,6^

* Authors contributed equally to this work.

^1^Trinity Centre for Biomedical Engineering, Trinity Biomedical Sciences Institute, Trinity College, Dublin, D02 R590, Ireland

^2^Dept. of Mechanical, Manufacturing, and Biomedical Engineering, School of Engineering, Trinity College Dublin, Dublin 2, D02 DK07, Ireland

^3^School of Pharmacy and Pharmaceutical Sciences, Trinity Biomedical Sciences Institute, and Trinity St. James’s Cancer Institute, Trinity College Dublin, Dublin 2, D02 R590, Ireland

^4^Smurfit Institute of Genetics, School of Genetics and Microbiology, Trinity College Dublin, College Green, Dublin, Ireland

^5^School of Pharmacy and Biomolecular Sciences (PBS), Royal College of Surgeons in Ireland (RCSI), Dublin, Ireland.

^6^Advanced Materials and Bioengineering Research Centre, Trinity College Dublin & RCSI, Dublin 2, D02 VN51, Ireland

Corresponding author: Prof. David Hoey, email: [dahoey@tcd.ie](mailto:dahoey@tcd.ie)

**Supplemental Methods**

**Metabolic activity**

Alamar blue (AB) assay was performed to assess cellular metabolic activity and was carried out according to manufacturer’s instruction (Invitrogen). Briefly, osteocytes and osteoblasts were cultured in growth media for 24h as previously described. Subsequently media was either replenished with fresh growth medium (Serum Full) or changed to serum free medium (Serum Deprived). After 24h of culture, media was removed, and cells were rinsed with PBS. AB assay was conducted to assess cells metabolic activity. The AB solution was prepared either in fresh growth medium or serum free medium (1:10 dilution), for the respective groups. Then, a final volume of 1 ml of AB solution was added to each well. Following 4h incubation at 37 °C, AB fluorescence was quantified at the respective excitation and emission wavelength of 560 nm and 590 nm.

**Supplemental Figures**

**
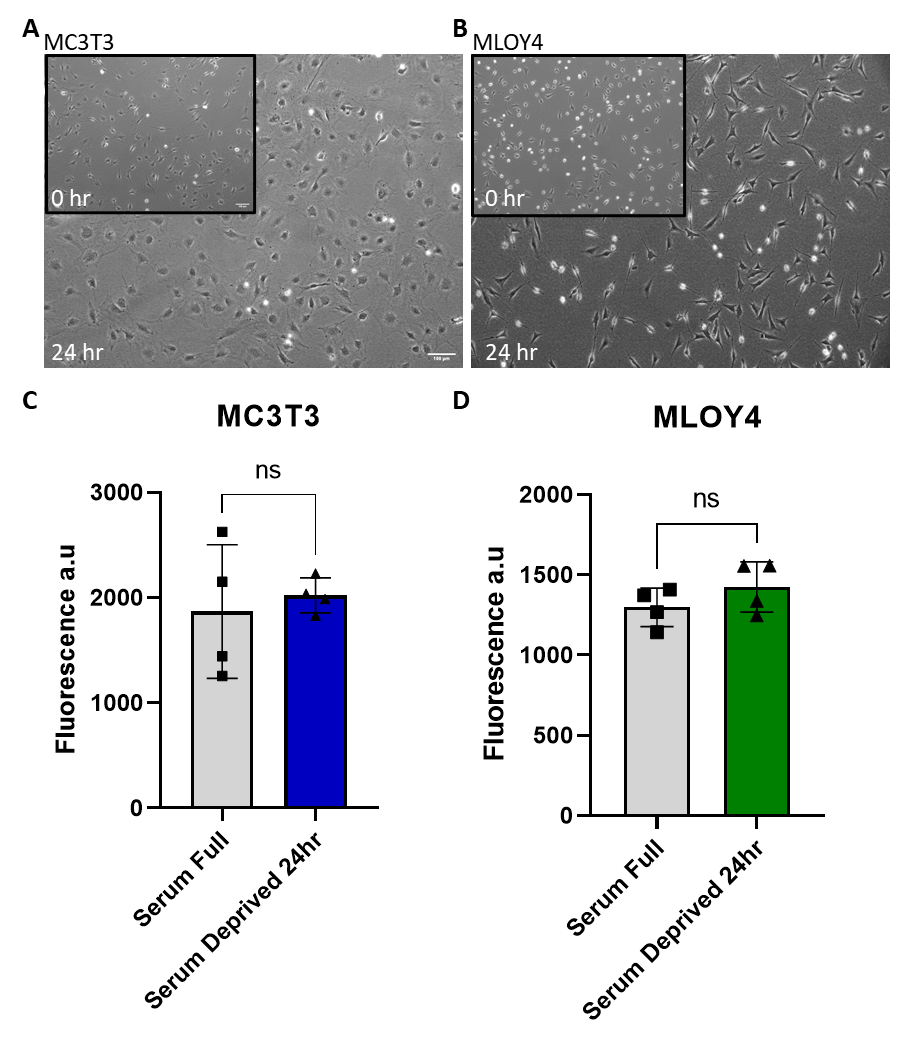
**

***Figure S1. (A)*** *MC3T3 and* ***(B)*** *MLOY4 cells demonstrate good viability following 24hrs culture in serum-deprived media. Scale bar: 100µm.* ***(C)*** *MC3T3 and* ***(D)*** *MLOY4 cells demonstrate no change in metabolic activity, as measured by Alamar Blue, following 24hrs culture in serum-deprived media when compared to cells cultured in standard growth media for same duration. Data presented as Mean ± SD, N = 4.*

**
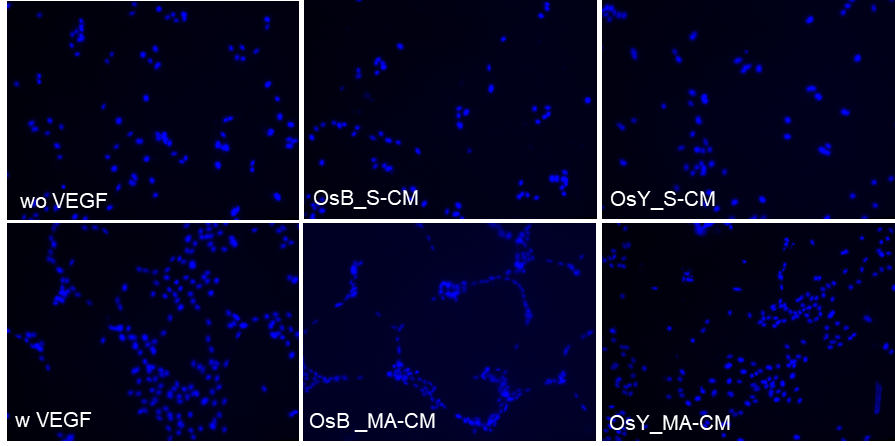
**

***Figure S2.*** *Representative images of DAPI stained HUVECs after 24 hours cultured in fresh medium without VEGF (negative control), with VEGF (positive control), statically (OsB_S-CM) or mechanically activated (OsB_MA-CM) MC3T3-E1 CM, and statically (OsY_S-CM) or mechanically activated (OsY_MA-CM) cultured MLOY4 CM*
